# Clinical presentation of axial myopathy in two siblings with HTLV-1 associated myelopathy/tropical spastic paraparesis (HAM/TSP)

**DOI:** 10.1186/s12883-015-0275-7

**Published:** 2015-02-28

**Authors:** Eiji Matsuura, Akiko Yoshimura, Satoshi Nozuma, Itsuro Higuchi, Ryuji Kubota, Hiroshi Takashima

**Affiliations:** Department of Neurology and Geriatrics, Kagoshima University Graduate School of Medical and Dental Sciences, 8-35-1 Sakuragaoka, Kagoshima, 890-8520 Japan; School of Health Sciences, Faculty of Medicine, Kagoshima University, 8-35-1 Sakuragaoka, Kagoshima, 890-8520 Japan; Center for Chronic Viral Diseases, Kagoshima University Graduate School of Medical and Dental Sciences, 8-35-1 Sakuragaoka, Kagoshima, 890-8520 Japan

**Keywords:** Axial myopathy, HTLV-1, HAM/TSP, Paraspinal muscle

## Abstract

**Background:**

The clinical features of myositis related with Human T-cell leukemia virus type 1 (HTLV-1) remains unclear despite epidemiological studies suggesting inflammatory myopathy associated with the virus.

**Case presentation:**

Here, we described the clinical presentations, muscle biopsy studies and laboratory results of two siblings with HTLV-1-associated myelopathy / tropical spastic paraparesis (HAM/TSP) who were affected with lumbar lordosis. Computed tomography (CT) scans demonstrated marked paraspinal muscle atrophy in both patients. Immunohistochemical studies of biopsy tissue obtained from one of the patients revealed inflammatory change of the muscle. Upon oral prednisolone therapy, the patient showed improvement in muscle strength and serum creatine kinase (CK) level.

**Conclusion:**

Myopathy or specifically axial myopathy should be considered as clinical symptom when treating the patients with HTLV-1 infection.

## Background

The human T-cell lymphotropic virus type 1 (HTLV-1) causes an inflammatory disorder of the central nervous system (CNS) termed HTLV-1-associated myelopathy/tropical spastic paraparesis (HAM/TSP) which is characterized by spastic paraparesis, urinary dysfunction, and paresthesia. While the neuropathology of HAM/TSP has been extensively described in the literature, the association between an HTLV-1 infection and muscular diseases are not well understood. HTLV-1 infection as a possible cause of myopathy was first noticed in early epidemiological studies demonstrating increased prevalence of viral antibody titer in patients with polymyositis (PM) from Jamaica and Japan where HTLV-1 is endemic. In 1989, Morgan reported that up to 85% of Jamaican patients with polymyositis (PM) tested positive for HTLV-1 antibody [1]. This observation was also confirmed by a subsequent study where HTLV-1 positivity was detected in 24/38 (63%) of Jamaicans patients with PM [2]. Additionally, Cupler et al. have suggested HTLV-1 as a cause of sporadic inclusion body myositis (sIBM) [3-5]. Japanese patient cohorts with either PM [6] or sporadic inclusion body myositis (sIBM) also had elevated frequency of HTLV-1 titer as compared to healthy individuals in the general population [7]. Previous pathological studies have demonstrated HTLV-1 localized to CD4+ perimysial lymphoid cells but not within muscle fiber cells [8,9]. However, some T-cell clonotypes, including one Tax11-19-specific clonotype were oligoclonally expanded among muscle-infiltrating lymphocytes [10]. The mechanisms of HTLV-1 related myositis in HAM/TSP patients are still sparse.

To broaden our understanding of HTLV-1 related diseases, we describe in this report the medical history, clinical characteristics and laboratory findings of two familial HAM/TSP patients with axial myopathy.

## Case presentation

### HAM/TSP diagnosis

Patient 1 was a 63-year-old female HAM/TSP patient with axial myopathy, a neuromuscular disease defined by weakness of spinal muscles. She has a family history of HTLV-1 infection and two older siblings, a brother who was diagnosed with HAM/TSP (73-year-old, Patient 2) and a sister who has been an asymptomatic carrier. Disease duration for both patients was approximately 23 years. Our records indicated that Patient 1 had a HTLV-1 proviral load of 414 copies/10000 PBMCs as well as virus-specific antibody response in serum (1:131072) and CSF (1:256); whereas, only serum HTLV-1 antibody data was available on Patient 2. Both patients exhibited progressive worsening of the lower extremities, spastic muscle tone of the legs, urinary disturbance, lack of sweating in the lower trunk and legs, marked hyperactive deep tendon reflex and pathological reflex such as Babinski’s, Chaddock’s reflexes. Bilateral paresthesia of the feet and keratoconjunctivitis sicca (dry eyes) was observed in Patient 1. MRI study revealed slight atrophy typical of HAM/TSP in thoracic spinal cord of Patient 1.

### Clinical feature of the siblings

**Figure 1:**
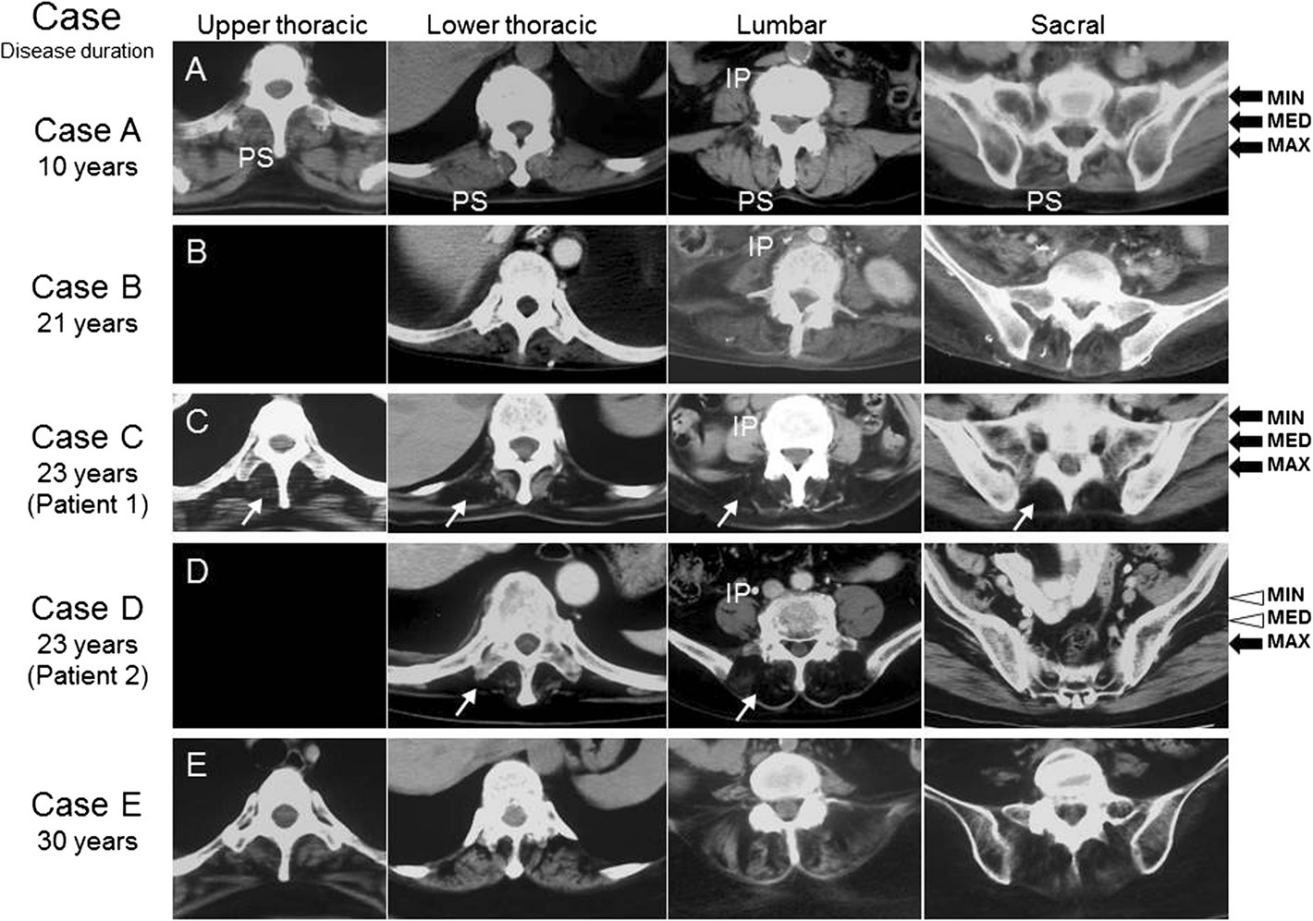
**Atrophy in paraspinal muscles of the siblings. (A-E)** Computed tomography studies of 5 patients with HAM/TSP at several truncal levels (upper thoracic, lower thoracic, lumbar and sacral levels). **(A, B, E)** The degree of muscle atrophy of the control HAM/TSP patients was in accordance with the duration of HAM/TSP. **(C, D)** The two siblings showed extreme atrophy of the paraspinal muscles (arrow) in comparison to the iliopsoas muscles (IP). The difference in atrophy between iliopsoas and paraspinal muscle of the two siblings is conspicuous in comparison to case B with similar disease duration. **(D)** The gluteus medius and minimus (arrow head) were also selectively atrophied in the elder brother. PS, paraspinal muscles; IP, iliopsoas; MAX, gluteus maximus; MED, gluteus medius; MIN, gluteus minimus.

In addition to HAM/TSP, the siblings were also afflicted with noticeable myopathy. Patient 1 has had a history of upper and lower back pains, she was admitted to the Orthopedics Department in her 40s at which time her serum creatine kinase (CK) level was significantly elevated at 5000 IU/L which was indicative of muscle damage. At a later hospitalization (62-year-old) due to recurrent urinary tract infections from intermittent self-catheterization, a Manual Muscle Testing (MMT) neuromuscular exam of her head, neck, upper and lower body was performed. She had normal responses in facial muscles but the weakness of neck flexion (MMT 4/5) and extension (4/5) as well as limitation of neck flexion. All of muscles groups in the upper extremities were normal (5/5) with the exception of deltoids (4/5) of both arms. Significant loss of muscle strength was observed in several muscle groups of her lower body including the iliopsoas (4/5), hip abductor (4/5), hip adductor (5/5), quadriceps femoris (4/5), hamstrings (3/5) tibialis anterior (5/5), and gastrocnemius (5/5) of both legs. Marked hyperactive deep tendon reflex and pathological reflexes are observed. Bilateral paresthesia of her feet but not hypoesthesia was noticed. No sweating was observed in the lower trunk and the legs. As such, the patient had difficulty standing without support of a guardrail and when attempting to maintain an erect orthostatic posture, she displayed a protruded belly and recessed back with anterior tilt of the pelvis that was characteristic of lumbar lordosis, an abnormal curvature of the spine. This clinical observation was further supported by electromyography (EMG) studies indicating fibrillation potentials and complex repetitive discharges in the thoracic paraspinal muscles as well as computed tomography (CT) scan demonstrating significant paraspinal muscle atrophy (Figure 1, Case C). Electrophysiological studies, such as a nerve conduction study (NCS), somatosensory evoked potential (SEP) and magnetic evoked potentials (MEP), were normal. A tissue biopsy was performed on her left deltoid muscle where immunohistochemistry study showed features of inflammatory myopathy and HTLV-1 provirus (the tax sequence, nucleotides 7358–7516) was also detected by PCR (data not shown).

Similarly, Patient 2 had a disease progression resembling that of his sister with visible paraspinal muscle weakness and lordosis as described above. He has had prior complaints (50 year-old) of muscle weakness of his extremities with an elevated serum CK level (>7000 IU/L). The CK level gradually normalized thereafter. At his age of 63, he experienced frequent urinary incontinence and was diagnosed as having uninhibited neurogenic bladder. He started to perform intermittent self-catheterization. Several years later, unlike Patient 1 who was able to walk with assistance, Patient 2 had become bed-ridden and had difficulty sitting or maintain an upright posture. At his age of 73, we evaluated his muscle strength and found normal muscular strength in his upper extremities (MMT 5/5) with the exception of the deltoid (4/5) muscles. Likewise, muscle groups in his lower extremities only demonstrated slight weakness and the results were as follow: iliopsoas (4/5), hip abductor (4/5), hip adductor (4/5), quadriceps femoris (4/5), hamstrings (4/5), tibialis anterior (5/5), and gastrocnemius (5/5) of both legs. CT scan demonstrated marked atrophy of paraspinal muscles and gluteus medius and minimus (Figure 1, Case D). Marked hyperactive deep tendon reflex and pathological reflexes are observed. There is no sensory disturbance but sweating was not detected in his legs.

### Muscle CT findings

We compared the two siblings described in this report (Table 1, Case C.D) with three of the control HAM/TSP patients (Table 1, Cases A.B.E). One (Case B) was a HAM/TSP patient with nearly the same duration of disease as the other two siblings, while the other two were the patients with shorter (Case A) and longer (Case E) durations of disease than the two siblings. The CT findings at several truncal levels revealed that the three control HAM/TSP patients (Figure 1, Cases A.B.E) gradually developed muscle wasting of the paraspinal muscles, iliopsoas and gluteus muscles in proportion to the duration of disease. In contrast, the two siblings (Case C.D) showed severe atrophy limited to the paraspinal muscles with well-preserved iliopsoas and gluteus maximus muscles. The elder brother (Case D) also showed severe atrophy of the gluteus medius and minimus, which helps to explain his difficulty in maintaining a standing posture.

**Table 1:** **Duration and status of the patients with HAM/TSP as shown in Figure** 1

**Case**	**Age**	**Sex**	**Duration of disease**	**Onset subject**	**Status**	**EDSS score**
A	67	M	10 years	Gait disturbance	Walking with stick	6.5
B	75	F	21 years	Gait disturbance	Bedridden	9.0
C	63	F	23 years (Case 1)	High back pain	Walking with stick	6.0
D	73	M	23 years (Case 2)	Low back pain	Bedridden	8.5
E	53	F	30 years	Gait disturbance	Wheelchair	7.0

### Blood work, genetic mutation analysis and treatment course of patient 1

Since myopathy in HAM/TSP patients is rarely described, we performed extensive laboratory analysis and whole genome molecular sequencing to rule out other causes of her disease. At the time of hospital admission (62-year-old), Patient 1 had a body mass index that was within the normal range (height = 159 cm; weight = 50 kg) and regular bowel movements. Except for a slightly elevated serum CK level of 187 IU/L (normal, 45–163 IU/L), CBC, urinalysis, total protein, electrolytes, bilirubin, cholesterol, alkaline phosphatase, lactate dehydrogenase, CRP, ESR, and thyroid hormone levels were unremarkable. Consistent with prior complaints of eye dryness and bilaterally positive Schirmer’s test, eye examination revealed superficial keratitis and corneal opacity. Other autoimmune connective tissue disorders such as rheumatoid arthritis and systemic lupus erythematosus as possible causes of her condition were excluded based on the test results of the autoantibody panel. Moreover, since the patient’s older brother (Patient 2) has spastic paraplegia and was also diagnosed with HAM/TSP, we questioned whether genetic mutation could be a contributing factor of their condition. DNA from Patient 1 was used in whole-exome analysis but no causative mutations were identified among the known disease-causing genes of inherited spastic paraplegia.

This patient was treated with oral prednisolone (50 mg/day) for three days. Upon treatment, she showed improvement in weakness and limitation of neck flexion, and weakness in deltoid and quadriceps femoris muscles. Although the abnormality in the curvature of her spine was somewhat reduced suggested by decreased protrusion of her belly following steroid treatment, her condition of lordosis was not completely reversed. She was then put on a tapered therapy with a10 mg/week decrease of prednisolone dosage and maintained a long-term therapy of 5 mg/day. During her follow-up examination, she was able to walk without a guardrail and her serum CK level was well within normal range at 32 IU/L.

### Muscle pathology and HTLV-1 detection

**Figure 2:**
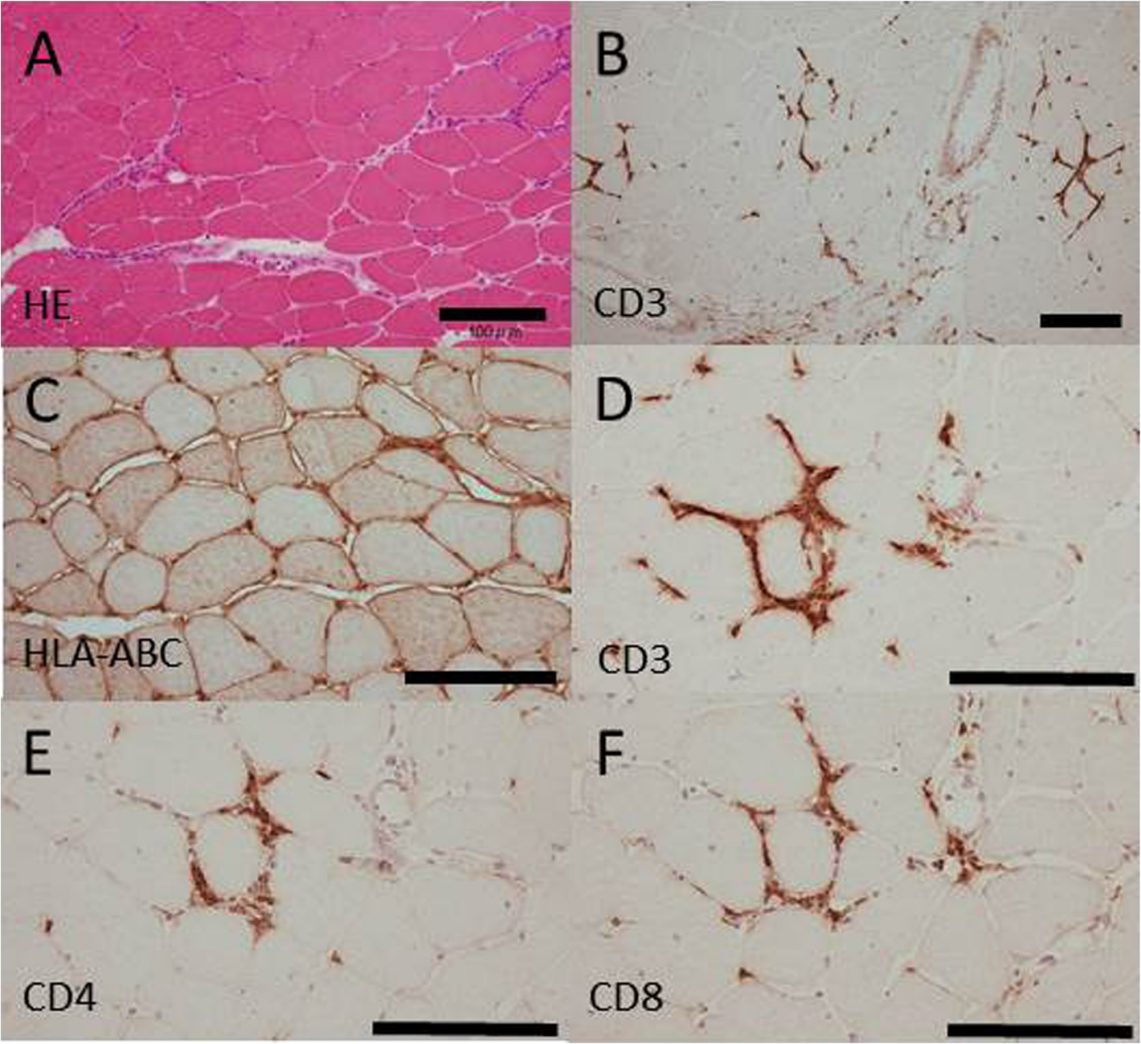
**Muscle biopsy.** Biopsy findings of the left deltoid muscle in Patient 1. **(A)** Hematoxylin and eosin staining. Mononuclear inflammatory cells were observed in the endomysium, perimysium and around the vessels. **(B)** Infiltrations of CD3-positive lymphocytes were occasionally observed in the endomysium. **(C)** An expression of HLA-ABC was markedly increased in the membrane of muscle fibers. **(D-F)** Sequential section of muscle tissue **(D)** A non-necrotic muscle fiber is surrounded by CD3+ lymphocytes. **(E, F)** The lymphocytic infiltrates were composed of CD4+ and CD8+ lymphocytes. Bar = 100 μm.

A biopsy of the left deltoid muscle in Patient 1 was performed and used for histochemical studies and HTLV-1 detection. A pathological study of the biopsied muscle revealed that muscle fibers ranged from 8 to 80 microns in diameter and had slight increase in the number of internal nuclei. Mononuclear inflammatory cells were observed in the endomysium, perimysium and around the vessels (Figure 2A). Occasional degeneration fibers having varying levels of oxidative enzyme activities was observed in the NADH tetrazolium reductase reacted sections. As judged from the ATPase reacted sections, the random checkerboard distribution of the histochemical fiber types was preserved. No rimmed vacuoles were observed with modified Gomori Trichrome staining. The acid phosphatase activity was increased in the invading macrophages. Immunohistochemical study revealed that CD4-positive lymphocytes and CD8-positive lymphocytes surrounding a non-necrotic fiber (Figure 2B) and elevated expression of HLA-ABC in the membrane of muscle fibers (Figure 2C). These findings are compatible with inflammatory myopathy.

In addition, DNA was extracted from the muscle (DNeasy tissue kit, QIAGEN, Tokyo, Japan) for virus detection. HTLV-1 SK43 and SK44 primers were used for tax DNA fragment. HTLV-1 provirus (the tax sequence, nucleotides 7358–7516) was detected by PCR, with some modification (Data not shown) [11].

### Genetic analysis for hereditary spastic paraparesis

Genomic DNA from the PBMC of Patient 1 was used in whole-exome sequencing analysis. Purified DNA was amplified using the Ion AmpliSeq Exome Kit (Life Technologies, Carlsbad, CA, U.S.A.), and sequenced on a Proton PI chip Version 2 (Life Technologies) according to the manufacturer’s protocol. Alignment and variant detection was performed using the Torrent Suite™ software with default parameters. No causative mutations were identified among the known disease-causing genes of inherited spastic paraplegia.

## Discussion

The most characteristic finding of the siblings with HAM/TSP was the remarkable wasting of the paraspinal muscles contrary to well-preserved iliopsoas muscle, gluteus muscles and the muscles of the extremities. Back pain as an initial symptom of the patient may suggest such condition. Paraspinal muscle atrophy to some degree is seen in various conditions such as amyotrophic lateral sclerosis, myasthenia gravis, muscular dystrophy, isolated extensor myopathy, sporadic inclusion body myositis, mitochondrial dysfunction, and axial myopathy. The immunohistochemical findings and fair improvements of her muscle weakness, limitation of neck flexion and serum CK level with oral prednisolone therapy strongly suggest they have both HAM/TSP and unique acquired inflammatory myopathy, although the possibility that the siblings may have unknown hereditary muscular diseases still remains.

Previous clinical or pathological studies have shown myopathic features in HTLV-1-infected individuals. Unique clinical manifestations were reported in HTLV-infected patients with inflammatory myopathy. Hokezu et al. also reported that the two sisters with myositis and HTLV-1 infection walked protruding their belly with a lordosis [12]. Littleton et al. reported HTLV-1-positive patient with rimmed vacuoles presenting with respiratory failure [13]. Honma et al. reported HAM/TSP patient with rimmed vacuoles presenting waddling gait [14]. Matsuura et al. reported that waddling gait was seen in 6 of 11 HTLV-1-positive patients with sIBM [7]. Inflammatory myopathy with HTLV-1 infection might show unique manifestation involving axial muscles in some cases. Although clinical symptom of the patients with HAM/TSP is spastic paraparesis, immunohistochemical studies on the muscles of HAM/TSP patients have revealed inflammatory or non-inflammatory myopathic change in over 50% of HAM/TSP patients [15,16]. The other study has shown that HTLV-1-positive patients with polymyositis had frequent cytochrome c oxidase deficiency and ultrastructural abnormalities in mitochondria of the affected muscle [17]. Sakiyama et al. reported the cases with a mitochondrial abnormality presenting axial myopathy, although the case was not infected to HTLV-1 [18]. Some patients with HTLV-1 infection and mitochondrial dysfunction might have association with axial myopathy, although any vacuoles, aggregates and mitochondrial pathology were not observed in biopsied muscle of our patient.

## Conclusion

Our patient with HAM/TSP showed a fair improvement of muscles’ weakness, limitation of neck flexion and standing posture with prednisolone therapy. Inflammatory myopathy or specifically axial myopathy should be considered as clinical symptom when treating the patients with HAM/TSP. A better understanding the site of lesion would lead to more appropriate medical treatment or physical therapy, resulting in delaying or minimizing the progressive neurological deficits seen in HAM/TSP patients.

### Consent

Written informed consents were obtained from the patients for publication of this Case report and any accompanying images. Copies of the written consents are available for review by the Editor of this journal.

### Ethics statement

This study was approved by the Institutional Review Boards of Kagoshima University.

## Abbreviations

HTLV-1: Human T-cell leukemia virus type 1
PM: Polymyositis
sIBM: Sporadic inclusion body myositis

## References

[1] Morgan OS, Rodgers-Johnson P, Mora C, Char G (1989). HTLV-1 and polymyositis in Jamaica. Lancet.

[2] Gilbert DT, Morgan O, Smikle MF, Simeon D, Barton EN (2001). HTLV-1 associated polymyositis in Jamaica. Acta Neurol Scand.

[3] Cupler EJ, Leon-Monzon M, Miller J, Semino-Mora C, Anderson TL, Dalakas MC (1996). Inclusion body myositis in HIV-1 and HTLV-1 infected patients. Brain.

[4] Ozden S, Gessain A, Gout O, Mikol J (2001). Sporadic inclusion body myositis in a patient with human T cell leukemia virus type 1-associated myelopathy. Clin Infect Dis.

[5] Ozden S, Cochet M, Mikol J, Teixeira A, Gessain A, Pique C (2004). Direct evidence for a chronic CD8 + −T-cell-mediated immune reaction to tax within the muscle of a human T-cell leukemia/lymphoma virus type 1-infected patient with sporadic inclusion body myositis. J Virol.

[6] Higuchi I, Nerenberg M, Yoshimine K, Yoshida M, Fukunaga H, Tajima K (1992). Failure to detect HTLV-I by in situ hybridization in the biopsied muscles of viral carriers with polymyositis. Muscle Nerve.

[7] Matsuura E, Umehara F, Nose H, Higuchi I, Matsuoka E, Izumi K (2008). Inclusion body myositis associated with human T-lymphotropic virus-type I infection: eleven patients from an endemic area in Japan. J Neuropathol Exp Neurol.

[8] Higuchi I, Hashimoto K, Kashio N, Izumo S, Inose M, Izumi K (1995). Detection of HTLV-I provirus by in situ polymerase chain reaction in mononuclear inflammatory cells in skeletal muscle of viral carriers with polymyositis. Muscle Nerve.

[9] Leon-Monzon M, Illa I, Dalakas MC (1994). Polymyositis in patients infected with human T-cell leukemia virus type I: the role of the virus in the cause of the disease. Ann Neurol.

[10] Saito M, Higuchi I, Saito A, Izumo S, Usuku K, Bangham CR (2002). Molecular analysis of T cell clonotypes in muscle-infiltrating lymphocytes from patients with human T lymphotropic virus type 1 polymyositis. J Infect Dis.

[11] Kwok S, Ehrlich G, Poiesz B, Kalish R, Sninsky JJ (1988). Enzymatic amplification of HTLV-I viral sequences from peripheral blood mononuclear cells and infected tissues. Blood.

[12] Hokezu Y, Higuchi I, Yanai S, Nagai M, Nagamatsu K (1994). [A family case of HAM and HTLV-I carrier including two sisters presenting with myositis]. Rinsho shinkeigaku = Clin Neurol.

[13] Littleton ET, Man WD, Holton JL, Landon DN, Hanna MG, Polkey MI (2002). Human T cell leukaemia virus type I associated neuromuscular disease causing respiratory failure. J Neurol Neurosurg Psychiatry.

[14] Honma S, Yamada K, Moriwaka F, Shima K, Tashiro K (1991). [A case of HTLV-1 associated myelopathy and adult T-cell leukemia, presenting unique muscle pathology including rimmed vacuole]. Rinsho shinkeigaku = Clin Neurol.

[15] Inose M, Higuchi I, Yoshimine K, Suehara M, Izumo S, Arimura K (1992). Pathological changes in skeletal muscle in HTLV-I-associated myelopathy. J Neurol Sci.

[16] Gabbai AA, Wiley CA, Oliveira AS, Smith R, Schmidt B, Nobrega JA (1994). Skeletal muscle involvement in tropical spastic paraparesis/HTLV-1-associated myelopathy. Muscle Nerve.

[17] Abdullah HM, Higuchi I, Kubota R, Matsuura E, Hashiguchi A, Abdelbary NH (2011). Histopathological differences between human T-lymphotropic virus type 1-positive and human T-lymphotropic virus type 1-negative polymyositis. Clin Exp Neuroimmunol.

[18] Sakiyama Y, Okamoto Y, Higuchi I, Inamori Y, Sangatsuda Y, Michizono K (2011). A new phenotype of mitochondrial disease characterized by familial late-onset predominant axial myopathy and encephalopathy. Acta Neuropathol.

